# Identification and engraftment of new bacterial strains by shotgun metagenomic sequence analysis in patients with recurrent *Clostridioides difficile* infection before and after fecal microbiota transplantation and in healthy human subjects

**DOI:** 10.1371/journal.pone.0251590

**Published:** 2021-07-12

**Authors:** Sandeep Verma, Sudhir K. Dutta, Elad Firnberg, Laila Phillips, Rakesh Vinayek, Padmanabhan P. Nair

**Affiliations:** 1 Division of Gastroenterology, Sinai Hospital, Baltimore MD, United States of America; 2 University of Maryland School of Medicine, Baltimore, MD, United States of America; 3 Johns Hopkins Bloomberg School of Public Health, Baltimore, MD, United States of America; 4 Noninvasive Technologies, Elkridge, MD, United States of America; University of Minnesota Twin Cities, UNITED STATES

## Abstract

**Background:**

Recurrent *Clostridioides diffícile* infection (RCDI) is associated with major bacterial dysbiosis and colitis. Fecal microbiota transplantation (FMT) is a highly effective therapeutic modality for RCDI. While several studies have identified bacterial species associated with resolution of symptoms in patients, characterization of the fecal microbiome at the bacterial strain level in RCDI patients before and after FMT and healthy donors, has been lacking. The aim of this study was to examine the ability of bacterial strains from healthy donors to engraft in the gastrointestinal tract of patients with RCDI following FMT.

**Methods:**

Fecal samples were collected from 22 patients with RCDI before and after FMT and their corresponding healthy donors. Total DNA was extracted from each sample and analyzed by shotgun metagenomic sequencing. The Cosmos-ID analysis platform was used for taxonomic assignment of sequences and calculation of the relative abundance (RA) of bacterial species and strains. From these data, the total number of bacterial strains (BSI), Shannon diversity index, dysbiosis index (DI), and bacterial engraftment factor, were calculated for each strain.

**Findings:**

A marked reduction (p<0·0001) in the RA of total and specific bacterial strains, especially from phylum *Firmicutes*, was observed in RCDI patients prior to FMT. This change was associated with an increase in the DI (p<0·0001) and in pathobiont bacterial strains from phylum *Proteobacteria*, such as *Escherichia coli O157*:*H7* and *Klebsiella pneumoniae UCI 34*. BSI was significantly lower in this group of patients as compared to healthy donors and correlated with the Shannon Index. (p<0·0001). Identification and engraftment of bacterial strains from healthy donors revealed a greater diversity and higher relative abundance of short-chain fatty acid (SCFA)-producing bacterial strains, including *Lachnospiraceae bacterium 5_1_63FAA_u_t*, *Dorea formicigenerans ATCC 27755*, *Anaerostipes hadrus*and others, in RCDI patients after FMT.

**Interpretation:**

These observations identify a group of SCFA-producing bacterial strains from healthy donors that engraft well in patients with RCDI following FMT and are associated with complete resolution of clinical symptoms and bacterial dysbiosis.

## Introduction

*Clostridioides difficile* infection (CDI) continues to be a rising public health problem in elderly and hospitalized patients, both globally as well as in Western countries. In the US, as many as 500,000 new cases and 14,000 deaths per year have been reported. It is estimated that the cumulative incidence of CDI globally is 323 cases per 100,000 population [1]. Although the global costs associated with management of CDI are unknown, the total amount is estimated in the billions of dollars per year, reflecting the associated chronic healthcare expenditures [2].

Approximately 70–80% of CDI patients respond to a single course of antibiotic therapy, while the remainder require repeated courses of expensive antibiotics and sometimes drastic measures such as fecal microbiota transplant (FMT). FMT has now become an accepted therapeutic modality for patients with recurrent *Clostridioides difficile* infection (RCDI) who have not responded to antibiotics and other therapies. Previous studies by our group and others have demonstrated that RCDI is associated with marked reductions in both the relative abundance (RA) of various bacterial communities and overall bacterial diversity [3, 4]. Most of these earlier studies were performed using 16S rRNA marker gene analysis, which identifies a specifically amplified target sequence of bacterial DNA rather than the entire genomic sequence which limits the identification of a bacterium to the genus level only. With application of this technology, several previous studies have reported that there is marked diminution of bacteria from families *Lachnospiraceae*, *Ruminococcaceae and Eubacteriaceae* in untreated RCDI patients [3–6]. This methodology is unable to assess sub-species (strain) level identification of the bacteria. The strain level identification requires high resolution deep shotgun metagenomic sequence analysis. A previous study by Simillie et al has recently reported application of shotgun metagenomic sequencing in RCDI patients before and after FMT, however this study did not report all bacterial sub-species involved [18]. Furthermore, the study design was different as 4 unpaired donors provided fecal sample for FMT to 19 RCDI patients. In addition, majority of post-FMT samples were collected in less than 4 weeks, raising question on issues about intraluminal detection of these bacterial strain vs their mucosal engraftment.

A high-resolution characterization of the human gut microbiome in patients with RCDI and their healthy donors is needed for a comprehensive and reliable insight of bacterial dysbiosis in RCDI and other GI disorders. To accomplish this, we have applied deep shotgun metagenomic sequence analysis to fecal samples collected from healthy donors and corresponding patients with RCDI before and after FMT. It is noteworthy that all the post-FMT fecal samples were collected 30 days or later from the FMT administration to evaluate engraftment of these bacterial strains. We have postulated that application of this technology may provide identification of unique bacterial strains with characteristic metabolomics and high engraftment capability.

## Material and methods

### Study design

This is an ongoing prospective, open-label, single-center study being conducted at Sinai Hospital of Baltimore. Twenty-two patients and 21 corresponding patient-selected donors were included in the study, with one donor providing fecal samples for two different RCDI patients. Eighteen RCDI patients and their corresponding donors brought their fresh fecal samples (same-day sample collection) to the endoscopy center on the day of the transplantation, as reported previously. Four patients received frozen fecal samples collected from donors prior to the day of FMT. Patient fecal samples were collected prior to starting the bowel preparation and sent to the microbiology lab, where they were aliquoted and frozen at -80C. Fresh donor samples were also sent to the microbiology lab, where they were homogenized, processed, and aliquoted. Aliquots of donor samples were sent to the endoscopy center, where they were transplanted in patients by endoscopy and colonoscopy as described previously [5]. Additional aliquots of each donor sample were stored at -80C. Patients were followed up in the GI clinic for up to two years, with post-FMT fecal samples collected at several timepoints. This study was reviewed and approved by the institutional review board of Sinai Hospital of Baltimore; IRB number 1826. Written informed consent was obtained from all study subjects, including both patients and patient-selected healthy donors.

### Patient selection

This study predates the incorporation of MDRO and COVID-19 guidelines issued by the FDA in 2020. Patients were referred to Sinai Hospital from area hospitals and were at least 18 years of age presenting with RCDI. RCDI was defined as three or more documented episodes of *C*. *difficile* infection with positive PCR and toxin assays occurring within 8 weeks of each other. All patients had been treated with commonly used antibiotics (Metronidazole, Vancomycin, Fidaxomicin) and had documented recurrence of CDI after discontinuation of antibiotic therapy.

### Donor selection

Donors were selected by the patients with RCDI (in all but four cases) and most were related to the patients. All donors were screened by a stringent protocol to exclude systemic or GI tract infections. Medical history was reviewed, and a physical examination was performed for each donor in the GI clinic. Blood and stool samples were collected and analyzed appropriately. Blood samples were analyzed for HIV, HAV, HBV, HCV, syphilis, HTLV1, and HTLV2; and stool samples were tested for the presence of *C*. *difficile* toxin and antigen, *H*. *pylori*, *Salmonella*, *Shigella*, *Yersinia*, *Campylobacter*, *Vibrio spp*, ova-parasite exam for *Giardia spp*., *Cryptosporidium spp*., *and Microspora spp*. Additionally, the presence of the *Clostridium difficile* toxin gene was determined by PCR. Stool collection kits with directions were provided to all study subjects and donors by the GI clinic.

### Donor exclusion

**Exclusion criteria included the following**.

1. Pregnant women or breastfeeding mothers.
2. History or presence of past or present infection with potentially infectious pathogens, including Human Immunodeficiency Virus; Hepatitis B; Hepatitis C; *Clostridium difficile* infection; enteric pathogens, including *Salmonella sp*., *Shigella sp*., *Campylobacter jejunii*, *Yersinia sp*., *Vibrio sp*., *Giardia lamblia*, *Cryptosporidium sp*, *and Microspora sp*.*; Helicobacter pylori*, *Treponema pallidum*, and Human T-cell Lymphotropic Virus 1 and 2.
3. Active malignancy or a history of cancer within the previous five years.
4. Antibiotic use or hospitalization within the past six months.
5. Recently hospitalized or discharged from a long-term care facility.
6. Abnormal laboratory results, including a positive test result for HIV, hepatitis B, or hepatitis C; *Helicobacter pylori* infection; gastrointestinal illness, including history or presence of peptic ulcer disease, gastroesophageal reflux disease, irritable bowel syndrome, inflammatory bowel disease, colonic polyps, colon cancer, cholecystitis, or diverticular disease; or renal, hepatic, hematologic, gastrointestinal, endocrine, pulmonary, immunologic, or systemic infection/infectious illness.

#### Fecal sample preparation and FMT procedure

Fecal sample preparation for FMT was conducted in the Microbiology Laboratory and/or Gastroenterology Research Laboratory at Sinai Hospital of Baltimore. A portion of each whole stool sample was saved and stored at -80 ^o^C for metagenomic analysis. Approximately 30-50g of stool were mixed with sterile saline and homogenized with a blender. This mixture was then passed through a series of filters to remove particulate material >250 microns. The final volume of the mixture was approximately 250ml. All processed fecal samples were administered to RCDI patients in the GI Diagnostic Center at Sinai Hospital of Baltimore. Twenty-five percent of the total fecal filtrate was delivered to the jejunum via enteroscope, and approximately 75% of the total fecal filtrate was delivered to the cecum and ascending colon via colonoscope in each patient. Successful therapy was defined as resolution of diarrhea and associated symptoms of RCDI following FMT. The detailed protocol for fecal filterate preparation and fecal sample storage is available online at Song Y, Garg S, Girotra M, Maddox C, von Rosenvinge EC, Dutta A, et al. (2013) Microbiota Dynamics in Patients Treated with Fecal Microbiota Transplantation for Recurrent *Clostridium difficile* Infection. PLoS ONE 8(11): e81330. doi:10.1371/journal.pone.0081330 (database).

#### Library preparation & analysis

Approximately 1.8 mls of frozen whole stool samples from donors and patients were sent to Admera Health, LLC (South Plainfield, NJ) for DNA extraction and library preparation. In brief, the gDNA was extracted using the NucleoSpin Soil kit (Macherey-Nagel) and the concentration of gDNA was assessed by the Qubit dsDNA High Sensitivity assay kit (Thermo Fisher). Libraries were prepared with the Nextera XT DNA Library Preparation Kit (Illumina) following the manufacturer’s protocol. The concentration of final libraries was measured by qPCR and equal amounts of DNA libraries were pooled, denatured with 0.2N NaOH, and then run on the NovaSeq S4, generating 20-100M 150-nt paired-end reads per sample. FastQ files were submitted to Cosmos ID for metagenomic analysis based on their Genbook database of 160,000 phylogenetically organized genomes and gene sequences [7–10]. This methodology uses a high-performance data-mining k-mer algorithm to disambiguate short sequencing reads into discrete genomes. The pipeline has two separable comparators: the first consists of a pre-computation phase for reference databases and the second is a per-sample computation. The input to the pre-computation phase consists of databases of reference genomes, virulence markers, and antimicrobial resistance markers. The output from the pre-computation phase is a phylogenetic tree, together with sets of variable-length k-mer fingerprints (biomarkers) uniquely associated with distinct branches and leaves of the tree. The second per-sample computational phase searches the hundreds of millions of short sequence reads against the fingerprint sets. The resulting statistics are analyzed to return fine-grain taxonomic and relative abundance estimates for the microbial NGS datasets. To exclude false positive identifications, the results are filtered using a filtering threshold derived from internal statistical scores that are determined by analyzing a large number of diverse metagenomes. The same approach is applied to enable the sensitive and accurate detection of genetic markers for virulence and for resistance to antibiotics. For each sample, species and strain relative abundance was calculated based on the number of organism-specific k-mers and their observed frequency in the sample, and normalized to represent the relative abundance of each organism. The CosmosID bioinformatics pipeline has been validated against the 35 benchmarking datasets from McIntyre *et al*. (CosmosID.com) and has been used in multiple previous studies [11–13]. Moreover, the bacterial strain detection specificity, sensitivity and precision of CosmosID’s algorithms may be more comprehensive than other available methodologies [11]. A comparative analysis of this methodology demonstrates better performance of this bioinformatic pipeline over 14 other tools for accurate identification of various bacteria as sub-species (strains) level. An entire benchmarking dataset is based on F1, precision, recall, and AUPR. (F1 score is the harmonic mean of recall and precision, weighting them equally in a single metric, AUPR is area under the precision-recall curve.) Additionally, we have applied a filtering threshold that retains only high confidence strain identifications and eliminates lower confidence ones that require further validation. Such filtering is primarily recommended for clinical application, where the samples that have high complexity in the phylogeny, abundance and environment. Furthermore, we also performed an average nucleotide identity (ANI) analysis using the JSpecies platform to evaluate the similarity of identified strains within a species

#### Shannon index

The Shannon index is a measure of bacterial species diversity, calculated as a function of the relative abundance of each species within a sample.

Shannon index *H* = ∑(*RA_i_*)ln(*RA_i_*) where RA_*i*_ is the relative abundance of species *i*

#### Dysbiosis and dysbiosis index

The compositional and functional alteration of the delicate balance of bacterial communities comprising the gut microbiome is termed dysbiosis. For each donor, pre-FMT, and post-FMT sample, dysbiosis was quantified using a dysbiosis index (DI), defined here as the ratio of the total number of *Proteobacteria* strains divided by the total number of bacterial strains per fecal sample.


$$\mathrm{DI}:\;\frac{\mathrm{Total}\;\mathrm{number}\;\mathrm{of}\;\mathrm{Proteobacteria}\;\mathrm{strains}}{\mathrm{Total}\;\mathrm{number}\;\mathrm{of}\;\mathrm{bacterial}\;\mathrm{strains}}$$

#### Bacterial engraftment and engraftment factor

Bacterial engraftment is a measure of the change in RA of a particular donor bacterial strain in the recipient’s gut microbiota following FMT. For each bacterial strain, the engraftment factor was calculated here as the ratio of (RA of the bacterial strain in the post-FMT minus RA pre-FMT) divided by RA in the donor.


$$\mathrm{Engraftment}\;\mathrm{Factor}:\;\frac{\mathrm{Post}\;\mathrm{FMT}\;\mathrm{RA}‐\mathrm{Pre}\;\mathrm{FMT}\;\mathrm{RA}}{\mathrm{Donor}\;\mathrm{RA}}$$

#### Statistical analysis and data tabulation

Standard statistical methods were used to assess differences between donor, pre-FMT, and post-FMT samples. A p-value threshold of <0·05 was used to assign statistical significance. The Shapiro-Wilk test was used to assess whether datasets were normally distributed. Non-normally distributed datasets (*Proteobacteria* and *Firmicutes* RA and # of strains) were compared using the non-parametric Kruskal-Wallis test with correction for multiple comparisons. All other datasets were compared by one-way ANOVA using the Tukey test with correction for multiple comparisons. The pictogram of the top 10 bacterial strains in Pre-FMT, Post-FMT, and donor groups was created using BioRender.com.

## Results

### Clinical features and clinical outcomes

The clinical characteristics of RCDI patients enrolled in the study are listed in Table 1. Mean patient age was 66 years, with 18 females and four males. The 21 donors had a mean age of 55 years, with nine females and 12 males. Donors were screened according to our protocol. The clinical outcome in patients with RCDI was defined as symptomatic resolution of *CDI*. Symptoms including diarrhea, bloating, abdominal pain, and cramping resolved in all patients within 3–7 days after FMT. Patients were followed up in the GI clinic for variable lengths of time with stool samples collected whenever possible, ranging from one week to two years post-FMT.

**Table 1:** Table detailing clinical characteristic of patients with RCDI.

Age (yrs)	Gender	Ethnicity	History of antibiotic use prior to C.diff	Symptoms	Comorbidities	Past Surgical history	Colonoscopy findings	Histopathology findings	Resolution of symptoms
**82**	F	C	+	diarrhea, abdominal pain, nausea	diverticulosis, HTN, DM, IBS	sigmoid colectomy	diverticulosis, hemorrhoids, polyp	Tubulovillous adenoma	Y
**41**	F	C	Clindamycin	diarrhea, abdominal pain, nausea, fever, chills	diverticulitis, HTN, IBS,	L hemicolectomy d/t severe diverticular disease	Colitis in ascending and transverse colon	mild acute and, focal active colitis	Y
**69**	M	C	Clindamycin	diarrhea, bloating	HTN, DM, COPD		moderately severe colitis	hyperplastic polyp in descending and sigmoid colon	Y
**63**	F	C	Clindamycin	diarrhea	IBS		normal	No significant pathologic changes	Y
**61**	F	C	+	diarrhea	HTN, DM, polyps	Polypectomy	Mild Colitis	Focal mild acute colitis in descending colon, acute colitis in rectum	Y
**44**	F	C	Ciprofloxacin	nausea, vomiting, abdominal pain, diarrhea	diverticulitis, HTN, IBS,	laproscopic sigmoid colectomy and colorectal anastamosis		No significant pathologic changes	Y
**78**	M	C	+	diarrhea, fever, abdominal pain	diverticulosis, HTN	Polypectomy	mild colitis, Mucosa appeared erythematous, edematous, and friable	mild active colitis in descending, sigmoid and rectum	Y
**59**	F	C	+	diarrhea, bloating, pain	IBS		Normal colon	no significant pathological changes	Y
**61**	F	C	Clindamycin	diarrhea, abdominal pain, cramping	HTN, diverticulosis	Polypectomy	Few scattered diverticula, 2 polyps in cecum	no significant pathological changes	Y
**91**	F	AA	+	diarrhea, abdominal discomfort, nausea, bloating, cramping	HTN, DM, diverticulosis	Polypectomy	multiple medium-sized scattered diverticula, polyp	no significant pathological changes	Y
**76**	F	C	+	diarrhea, abdominal discomfort, nausea	HTN, DM	Appendectomy, polypectomy	severe colitis	sigmoid colon- active colitis w/surface ulceration and associated fibrinopurulent exudate	Y
**71**	F	AA	+	diarrhea		Sigmoid resection for diverticular disease, polyps	mild colitis in cecum, ascending and descending colon—mucosa appeared erythematous and smooth	No significant pathologic changes	Y
**63**	F	C	Clindamycin	diarrhea, abdominal cramps	HTN		Normal colon	no significant histopathologic changes	Y
**79**	F	AA	+	diarrhea	HTN, DM, diverticulosis	Polypectomy	mild colitis	focal acute nonspecific colitis in ascending colon	Y
**57**	M	C	+	diarrhea	HTN, IBS, diverticular disease, diverticulitis		mild colitis, mucosa appeared erythematous and smooth, multiple scattered diverticula	Mild active colitis in cecum	Y
**22**	F	C	Levaquin	diarrhea, abdominal cramps	N/A	N/A	Mild colitis, mucosa appeared nodular	focal active colitis in rectum, negative for dysplasia	Y
**61**	F	AA	Ciprofloxacin	diarrhea, bloating, abdominal pain, nausea, blood in stool	HTN	Polypectomy	Mild colitis; Mucosa appeared edematous, erosive, erythematous, friable and thickened. Polyp in descending colon	Focal active colitis in descending and sigmoid colon, mild acute nonspecific colitis in rectum	Y
**71**	F	C	+	diarrhea, nausea, abdominal pain	IBS	Appendectomy	Moderately severe diverticulosis of sigmoid colon	Sigmoid colon: colonic mucosa w/mild congestion of lamina propria.	Y
**70**	F	C	Clindamycin	diarrhea	HTN, diverticulosis		few scattered diverticula	no significant pathologic changes	Y
**68**	F	AA	Clindamycin	diarrhea, abdominal pain	HTN, COPD	Polypectomy	Diffuse mild inflammation in sigmoid, descending, transverse and ascending colon.	Focal active colitis in ascending, descending, sigmoid and rectum.	Y
**79**	M	C	+	diarrhea	HTN, COPD, diverticulosis	Polypectomy	Multiple small and large mouthed diverticula—no evidence of bleeding.	Colonic mucosa w/no significant pathologic changes in ascending colon, descending colon and rectum	Y
**87**	F	AA	Clindamycin	diarrhea, abdominal pain	HTN, diverticulosis		Patchy mild inflammation in sigmoid and ascending colon	Acute nonspecific colitis in ascending, descending colon and rectum: mild to moderate acute colitis.	Y

AA: African American, C: Caucasian, M: Male gender, F: Female Gender, Y: Yes, +: Other antibiotics., IBS: Irritable Bowel Disease, IBD: Inflammatory Bowel Disease, COPD: Chronic Obstructive Pulmonary Disease, HTN: Hypertension, DM: Diabetes Mellitus.

### Bacterial diversity and Bacterial Strain Index (BSI)

**Fig 1:**
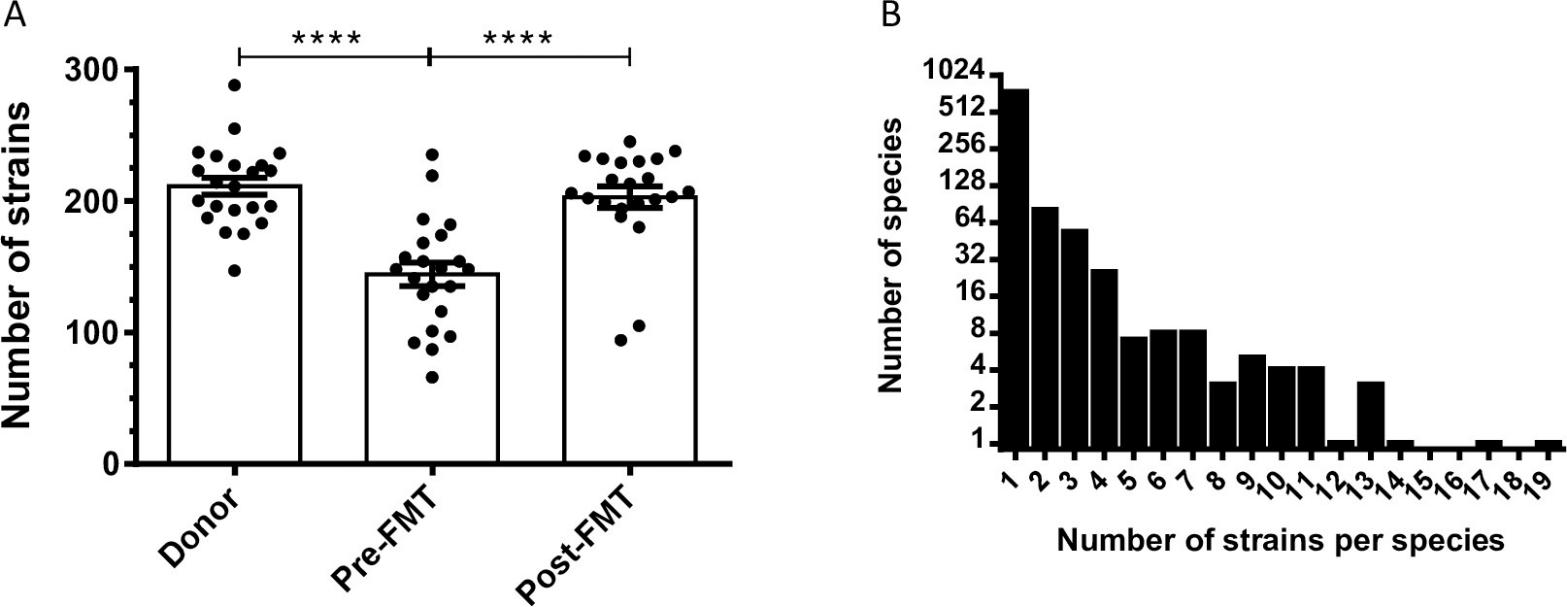
**A)** Scatterplots of total number of bacterial strains (BSI) in fecal samples from healthy donors, pre-FMT and post-FMT patients. A statistically significant difference (p<0.0001) was observed between donors and pre-FMT samples as well as between pre-FMT and post-FMT samples. **B)** Histogram depicting the number of bacterial strains per species of bacteria identified across all samples.

**Fig 2:**
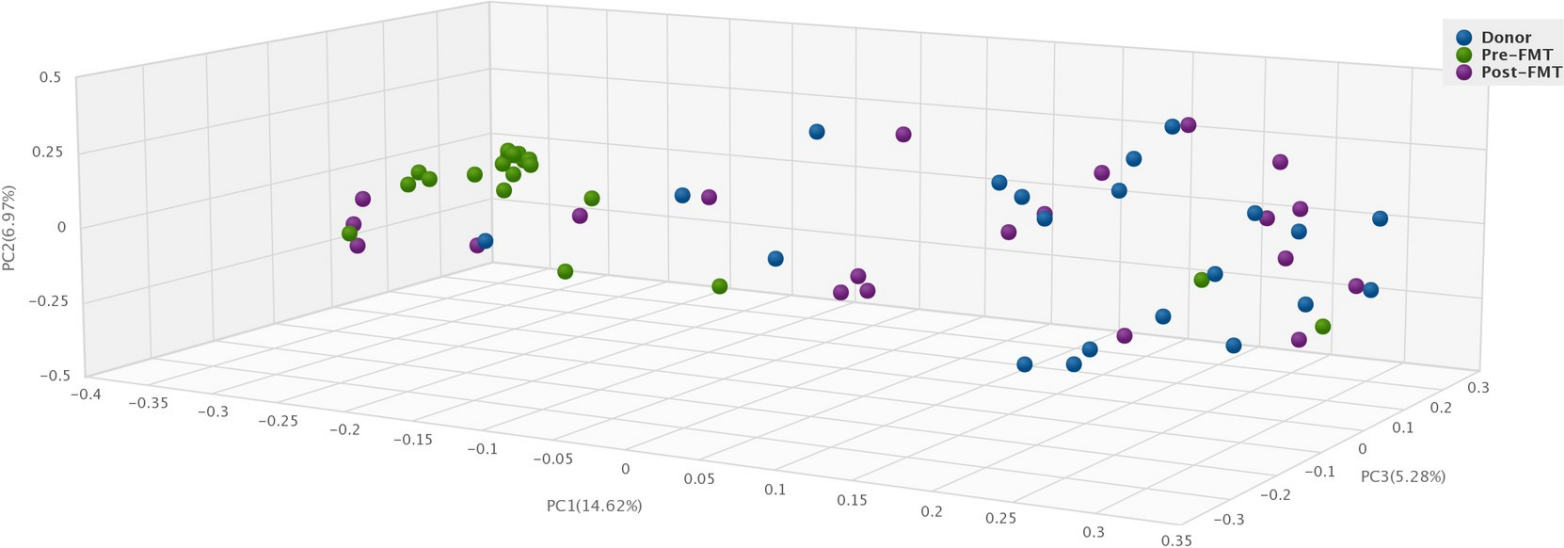
Beta diversity analysis using Bray-Curtis principle coordinate analysis demonstrates greater clustering of donor and post-FMT samples compared to pre-FMT samples.

**Fig 3:**
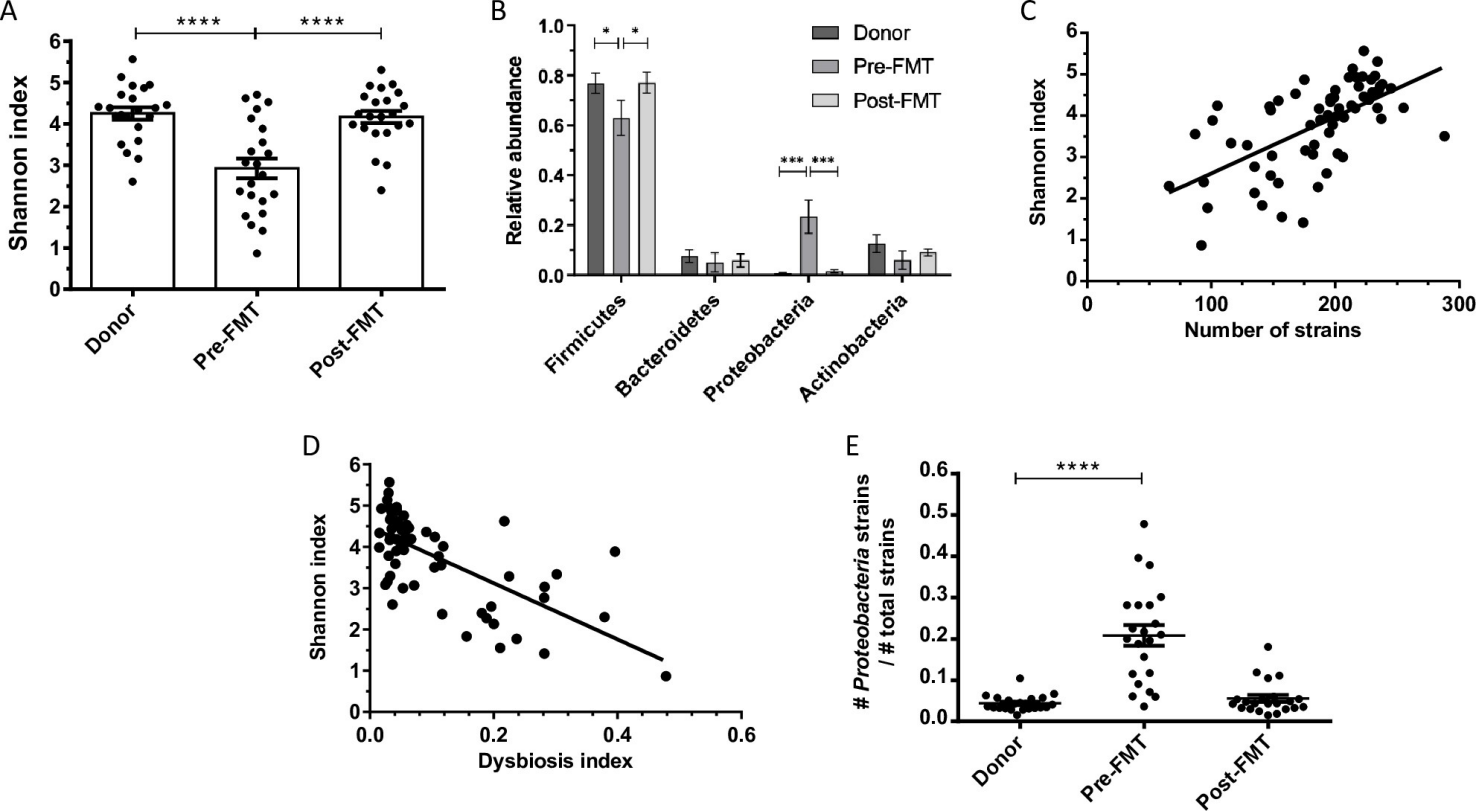
**A)** Column scatterplot of bacterial diversity expressed as Shannon Index (mean +/- SEM) in fecal samples obtained from healthy donors, pre-FMT and post-FMT patients. A statistically significant difference (p<0.0001) was observed between donor and pre-FMT samples as well as between pre-FMT and post-FMT samples. **B)** Relative abundance of four major phyla: *Firmicutes*, *Actinobacteria*, *Bacteroidetes and Proteobacteria* in fecal samples from healthy donors, pre- and post-FMT patients. There was statistically significant difference in *Firmicutes* between donor/post-FMT and pre-FMT samples (p<0.05) and a larger difference in *Proteobacteria* between donor/post-FMT and pre-FMT samples (p<0.0005). **C)** Scatterplot of Shannon Index vs. bacterial strain index (BSI). The linear regression Pearson correlation coefficient is r = 0.6158, r^2 = 0.3792 and the p-value is <0.0001 for non-zero slope. **D)** Scatterplot between dysbiosis index (DI) and Shannon Index. The linear regression Pearson correlation coefficient is r = -0.6737, r^2 = 0.4539, p-value < 0.0001 for non-zero slope. **E)** Column scatterplot of dysbiosis index (mean +/- SEM) in fecal samples in healthy donors, pre-FMT and post-FMT patients. A statistically significant difference (p<0.0001) was observed between donor/post-FMT and pre-FMT samples.

The total number of bacterial strains in each fecal sample was calculated based on shotgun metagenomic sequence analysis. The average number of bacterial strains per sample in the healthy donor group was 212·8 ± 31·86 (Fig 1A). The average number of bacterial strains (per sample) in pre-FMT samples from patients with RCDI was significantly lower, 144·2 ± 40·6 (p<0·001). Interestingly, after FMT, the average number of bacterial strains per sample increased significantly to 193·3 ± 51·9 (p<0·001) in this group of patients. Each bacterial species was represented by 1–19 strains, with a mean value of 1·64 (+/- 1·84 SD) strains per species (Fig 1B). The total number of bacterial strains per sample is expressed as bacterial strain index (BSI) and illustrated in a histogram (Fig 1A). We also observed that pair-wise comparison of 4 strains of *Clostridioides difficile*, or 15 strains of *Bifidobacterium longum* all had an ANI of >95%. Furthermore, to mathematically calculate compositional dissimilarity in various microbial communities present in the donor, pre-FMT, and post-FMT samples, we have applied a principle coordinate analysis using Bray-Curtis dissimilarity (Fig 2). Principle coordinate 1 (PC1) was highly associated with dysbiosis, as donor/post-FMT samples and pre-FMT samples formed distinct clusters along this axis. Additionally, to compare bacterial strain diversity to species diversity, we calculated the Shannon index, an indicator of species diversity, for each fecal sample. The mean Shannon index in the pre-FMT group was 2·92 ± 1·09 which was lower as compared to the healthy donor group 4·25 ± 0·67; and in the post-FMT group 4·18 ± 0·67. The differences in Shannon indices between pre-FMT and post-FMT groups and between pre-FMT and healthy donor groups were statistically significant (p<0·0001) (Fig 3A). As expected, there was a statistically significant correlation between BSI and Shannon index (Pearson correlation coefficient value = 0·61; p<0·0001) (Fig 3C). These findings suggest that BSI maybe a predictor of diversity as well and a tool to quantify bacterial strains.

### Dysbiosis Index (DI)

The mean DI (+/- SD), a measure of *Proteobacteria* abundance, was significantly higher in the pre-FMT group (0·22 +/- 0·38) than in healthy donors (0·003+/- 0·005; p<0·0001). The DI was lowered in patients after FMT, approaching the value in healthy donors (0·01+/-0·02) (p<0·0001).(Fig 3E). Furthermore, we observed a significant inverse correlation between the Shannon index and the DI in patients before and after FMT (Fig 3D)

### Recipient pre-FMT microbiota profiling

**Fig 4:**
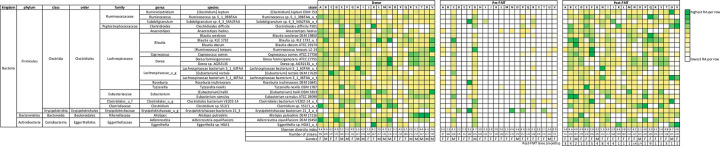
Heat map depicting the top 25 bacterial strains according to relative abundance in the healthy donor group. Shannon diversity index and number of bacterial strains (BSI) in each fecal sample are depicted at the bottom of the heat map.

**Fig 5:**
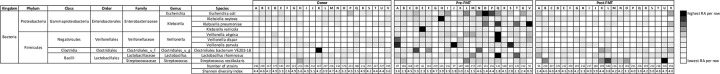
Heat map depicting the top 10 bacterial species sorted by highest relative abundance in the pre-FMT RCDI patient group. Shannon diversity index and number of bacterial strains are depicted at the bottom of heat map. A marked increase in RA of certain pathobionts was observed in the pre-FMT state as compared to donors and post-FMT samples.

**Fig 6:**
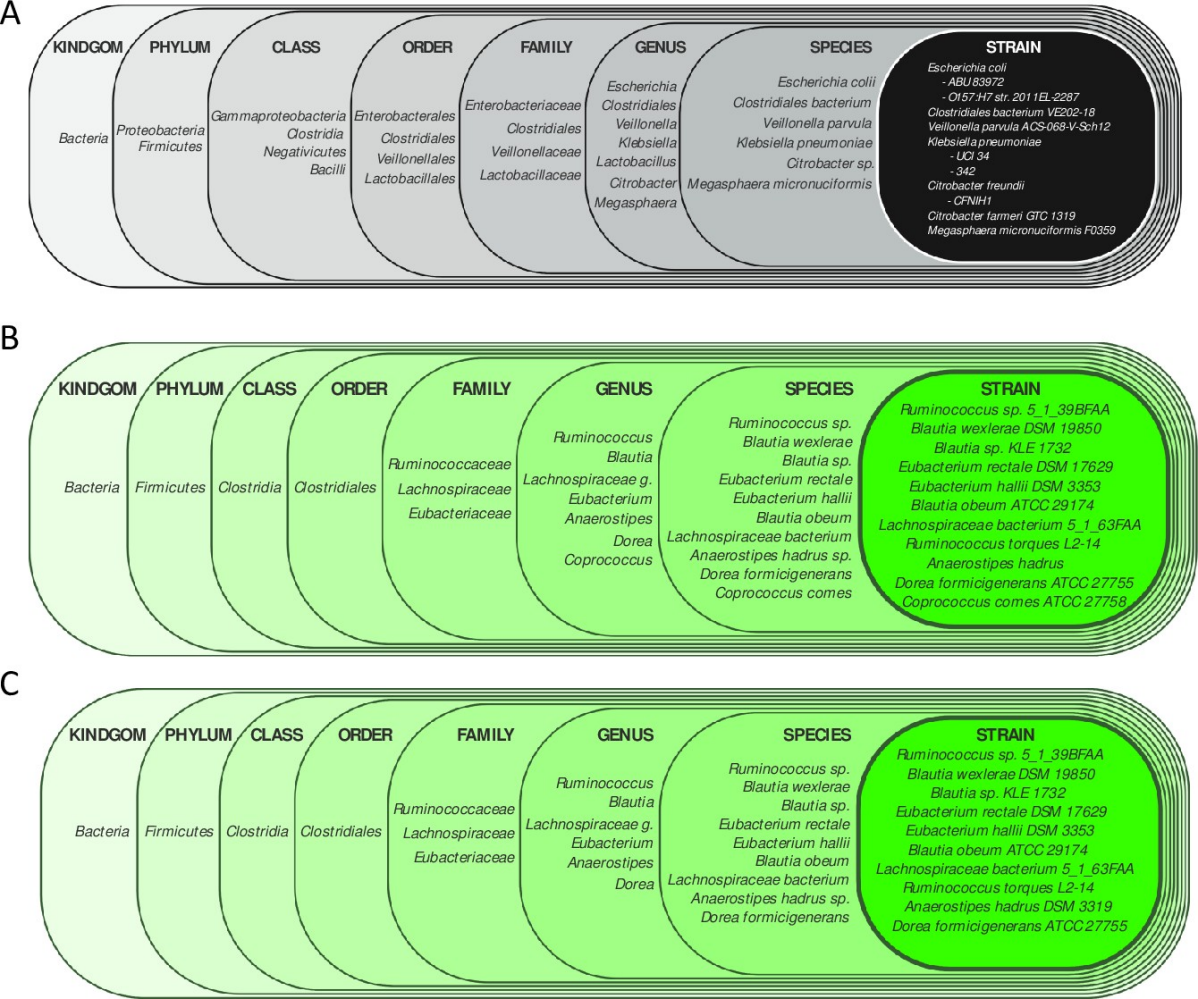
**A)** Pictogram depicting the 10 most abundant bacterial strains in **A)** pre-FMT RCDI patients, **B)** healthy donors, **C)** post-FMT RCDI patients.

We identified the 25 bacterial strains with the highest RAs in healthy donors and observed their abundance in patients with RCDI. All of these strains had markedly lower RAs in RCDI patients prior to FMT than in their corresponding healthy donors (Fig 4). The top ten bacterial strains by relative abundance in pre-FMT patient fecal samples were found to be predominantly pathobiont bacterial species of phylum *Proteobacteria* (Figs 5 and 6A).

### Recipient post-FMT microbiota profiling

Beta diversity analysis showed striking similarity between the fecal microbiota composition of healthy donors and post-FMT (Fig 2). Of the 25 most abundant strains in donors, 11 were present on average in pre-FMT patient samples which increased to 21 at their post-FMT follow-up visit sample, an indicator of engraftment (Fig 4). Post-FMT patients also displayed a marked diminution in the RAs of pathobionts (Fig 5). The top ten bacterial strains by RA isolated from post-FMT fecal samples were identical to those isolated from healthy donors (Fig 6B and 6C).

### Longitudinal microbiota profiling

**Fig 7:**
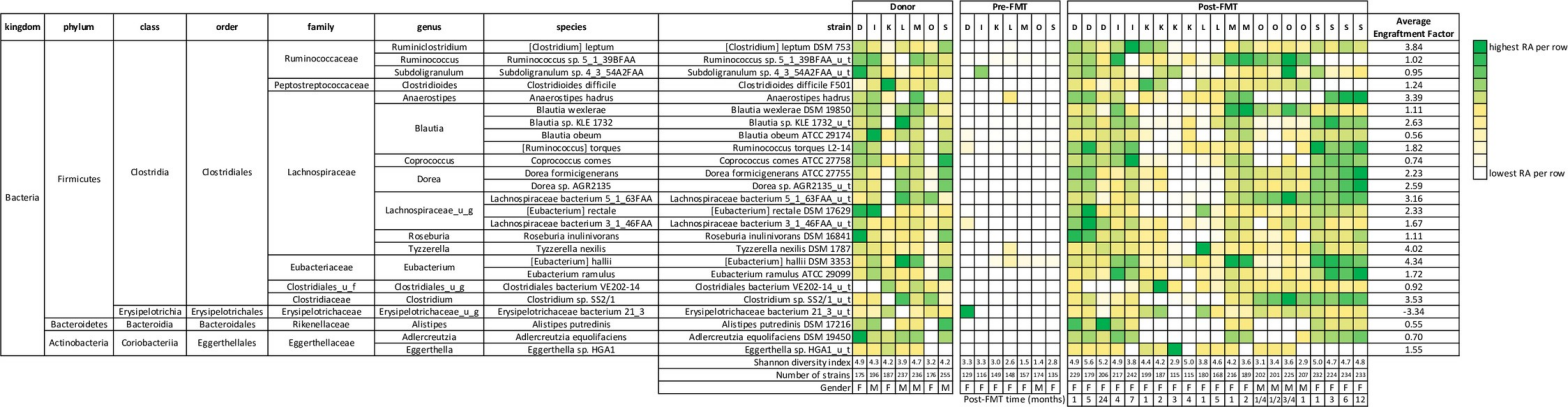
Heat map depicting the longitudinal mapping of the top 25 strains according to the relative abundance in the post-FMT group. The bottom of the heat map indicates sample collection time point in months post-FMT, Shannon index and total number of bacterial strains. Engraftment of bacterial strains from corresponding healthy donors was noted in patients with RCDI after FMT.

**Fig 8:**
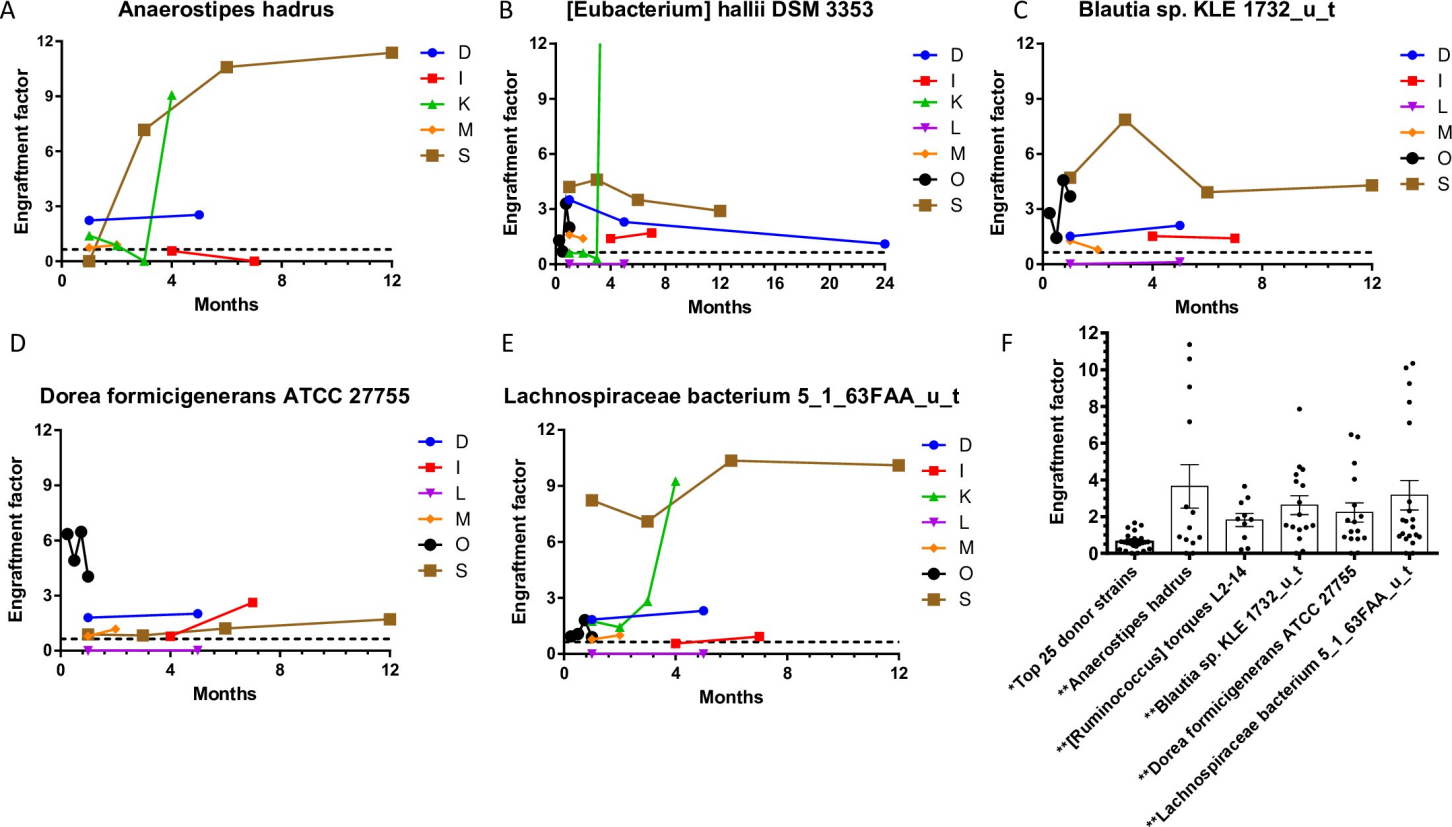
Longitudinal mapping of the engraftment factor of the top 5 engrafting strains in post-FMT patients, **A)** *Anerostipes hadrus***, B)** *[Eubacterium] halli DSM 3353*, **C)** *Blautia sp*. *KLE 1732_u_t*, **D)** *Dorea formicigenerans ATCC 27755*, *and* **E)** *Lachnospiraceae bacterium 5_1_63FAA_u_t*. **8F)** Column scatterplot depicting engraftment factor values for the top 5 engrafted bacterial strains from healthy donors in patients with RCDI after FMT as compared to the mean engraftment factor value of top 25 bacterial strains.

Longitudinal follow-up of up to two years was performed in seven FMT recipients, no previous study has provided microbiota to strain level for period extending beyond 30 days. The RAs of the top 25 donor strains are mapped in these patients across variable time periods (Fig 7). In each case, the overall strain composition of post-FMT fecal samples from a given patient resembles that of the corresponding donor sample and is strikingly different from the pre-FMT state. We followed five bacterial strains with the highest level of engraftment in these seven patients over varying time periods following FMT (Fig 8A–8F). The persistence of donor bacterial strains after a period of time exceeding two months was considered long-term engraftment. We were able to observe long-term engraftment of some (but not all) strains in each patient. Engraftment of each of these five strains in all 22 patients immediately following FMT is depicted in Fig 8F.

## Discussion

This unique study provides an in-depth examination and describes strain-level identification of human fecal microbiota by next-generation shotgun metagenomic sequence analysis in patients with RCDI and their corresponding patient-selected healthy donors. Strain-level analysis of the fecal microbiota allows us to measure a) the presence of specific bacterial strains in RCDI patients prior to FMT, and b) engraftment of specific bacterial strains from healthy donors following FMT. In addition, this type of analysis provides an opportunity to study the putative role of specific bacterial strains in RCDI patients prior to FMT and their possible role in resolution of bacterial dysbiosis by FMT. From a genomic point of view, a bacterial species is defined as a relatively homogenous population sharing over 97% 16S rRNA gene sequence similarity as agreed upon by the International Committee on Systematic Biology [14, 15]. With the advent of next generation sequencing (NGS) technology and bioinformatics, it has been possible to identify multiple strains of a single bacterial species as independent taxonomic entities. Although these strains share the same core genome, they may differ among themselves by as much as 30% in gene content and may have tremendous metabolic and functional diversity [16, 17].

In our study population, we identified 944 species and 1453 bacterial strains across all fecal samples. The number of bacterial strains per sample was significantly lower (p<0·001) in patients with RCDI before FMT as compared to healthy donors and increased markedly (p<0·001) after FMT (Fig 1A). Similar observations were reported in a study by Smillie et al., that aligned shotgun metagenomic reads from 31 gene markers to a database of 649 reference genomes in order to assign bacterial taxa as mg-OTUs (metagenomic Operational Taxonomic unit, roughly corresponding to a species) [18]. For identifying strains, they performed SNP(single nucleotide polymorphism) analysis within these 31 loci [18]. This study detected 306 mg-OTUs and 1091 strains across fecal samples from four healthy donors and 19 RCDI patients before and after FMT [18]. In the study by Simillie et al., while taxonomic units were classified phylogenetically by species and arbitrary strain identifiers assigned, the specific bacterial type strains represented by these units were not labelled by approved list of bacterial nomenclature published by International Journal of Systematic and Evolutionary Microbiology (IJSEM) [18]. It is noteworthy that in our study we used an algorithm that aligns the complete shotgun metagenomic read data across 160,000 reference genomes to identify the specific bacterial strains. Furthermore, the mean depth of sequence analysis in in the paper by Simillie et al was 1.3 x 10^10^ base pair (bp) as compared to our study where it averaged 1.1 x 10^10^ bp per sample. Additionally, in the study by Smillie at al., eight of the 79 samples were truly beyond 28 days and majority of samples were before 14 days, suggesting these may be intraluminal bacterial strain and no mucosal engraftment [18]. In contrast, in our study 22 RCDI patients received only one FMT from 21 patient selected donors and fecal samples were collected only after 30 days for the analysis. We have assumed donor strains from these samples represent true engraftment rather than intraluminal detection of bacteria. It is noteworthy that in our study 22 patients with RCDI received only one FMT as compared to the study by Smillie et al, where 8 of the 19 RCDI patients received an additional FMT within 14 days of the first administration of FMT. Another recent study by King et al., identified only 150 bacterial strains across 48 fecal samples from 16 healthy human subjects by NGS, however, this study did not include patients with RCDI [19]. We suspect that the number of strains in our study maybe higher due to greater depth of reads (20–100 million) and larger database (Genbook). The algorithm for identification of a bacterial strain in our study does not assemble full genomes de novo, however it does match reads (broken into kmers) to its reference strain genome database and assigns the unique and total kmer matches for each organism. Using deep shotgun sequence analysis, we detected from 1 to 19 strains per bacterial species in our study samples (Fig 1B), with a mean of 1·64 (+/-1·84) strains per species. We also examined strain genomic similarity using JSpecies platform for strains of Clostridioides difficile and fifteen strains of Bifidobacterium longum, they all demonstrated >95% genomic similarity. Different strains of a bacterial species can have vastly different metabolomics and functionality. For example, one strain of *Lactobacillus casei* (UW-4) is used for cheese production, another (A2-309) is associated with wine production, and a third (L3) is a commensal in the human GI tract [20]. In fecal samples from healthy donors, the 25 most abundant strains came from phyla *Firmicutes*, *Bacteroidetes*, and *Actinobacteria* (Fig 4). Members of these phyla are remarkable for their ability to produce short-chain fatty acids, which have been shown to feed colonic epithelial cells while inhibiting the growth and virulence of pathogens in the gut [21]. In RCDI patients who received chronic antibiotic therapy prior to FMT, the nine most abundant strains included six pathobionts from phylum *Proteobacteria* (Fig 5). Our study represents a comprehensive attempt to identify bacterial strains in the gut of RCDI patients and their corresponding healthy donors. However, the data should be interpreted in light of the limited completeness of reference genome databases, bioinformatics, and unmatched sequences.

Earlier studies expressed the diversity of microbial communities in human fecal samples by the Shannon index, a measure of species diversity. Instead, we use the bacterial strain index (BSI), which measures strain diversity. Similar to previous reports, the mean Shannon index was lower in our RCDI patients than in healthy donors before treatment and increased significantly after FMT (P<0·001, Fig 3A). Similarly, the mean BSI was lower in patients with RCDI before FMT and increased significantly after FMT, resembling BSI in the donor group (Fig 3A) (p<0·001). As expected, we found a significant correlation between BSI and Shannon Index, suggesting that BSI may be a good indicator of microbial diversity at the strain level (Fig 4).

The gut microbiome in healthy human subjects is mainly composed of four phyla: *Firmicutes*, *Bacteroidetes*, *Actinobacteria* and *Proteobacteria* [22]. In our study, *Firmicutes* was the most abundant phylum in healthy donors, followed by phyla *Actinobacteria*, *Bacteroidetes*, and *Proteobacteria* (Fig 3B). The high relative abundance of phylum *Firmicutes* in healthy donors may be attributable to stringent screening and exclusion criteria in our study, which included the absence of antibiotics for six months prior to fecal sample collection. Antibiotic use and dietary factors have been associated with an increase in the relative abundance of phylum *Bacteroidetes* in healthy human subjects [23]. It is noteworthy that in patients with RCDI, all of whom had received antibiotic therapy prior to FMT, the RAs of these four phyla differed markedly from those in healthy donors (Fig 3B). A significantly higher relative abundance of strains from the phylum *Proteobacteria* (23·3%) was noted in this group of patients compared to their corresponding healthy donors, in whom less than 1% of bacteria derived from this phylum (Fig 3B). A close examination of the bacterial strains belonging to the phylum *Proteobacteria* in the pre-FMT samples from patients with RCDI revealed an increase in the relative abundance of several pathobiont strains (Figs 5 and 6A). The word pathobiont refers to bacterial strains from various phyla, which can bloom and cause disease during the state of dysbiosis. C. difficile can also be considered a pathobiont, as it colonizes 17.5% of adults, with even higher rates seen in individuals having contact with the health system (hospitals, clinics, nursing homes, etc). These bacterial strains may become pathogenic under certain environmental factors (i.e. dietary, insecticide, pesticide, and antibiotics) and intraluminal conditions of the host (pH, bile acids, SCFA, mucous layer, and microbiota) [24, 25]. We identified pathobionts from the phylum *Proteobacteria* in pre-FMT RCDI patients (Fig 6A), including *E*. *coli O157*:*H7*, *Klebsiella pneumoniae UCI 34*, and *Veillonella parvula ACS-068-V-Sch12*. Recently, Atarashi et al have reported that certain pathobionts can induce T-helper 1 cells as well as inflammation in the human intestine [26]. In addition, Buffie *et al*. showed that a single dose of clindamycin can result in expansion of *Proteobacteria* in the intestinal microbiome of mice, which is accompanied by an increase in susceptibility to *C*. *difficile* colitis when *C*. *difficile* spores were inoculated in this group [27]. In a study of the long-term effects of vancomycin on the human intestinal microbiome, Isaac *et al*. noted a reduction in microbiota diversity and expansion of phylum *Proteobacteria*, accompanied by an increase in the relative abundance of *Escherichia/Shigella* and *Klebsiella–*a profile similar to that of our RCDI patients before FMT [28].

In our study, we detected 6 different strains of C. difficile in fecal samples: F501, P51, T42, 840, M120 and P28. BLAST search of these strain genomes and proteomes revealed that strains F501, P51 and P28 lack the tcdA/tcdB toxin genes or cdtA/cdtB binary toxin genes and are non-pathogenic. Strains T42, M120 and 840 present in Pre-FMT samples do carry these toxin genes and are considered toxigenic strains. In twelve out of 22 RCDI patients prior to FMT, only C. difficile strains P28 (n = 5), F501 (n = 4), T42 (n = 1), 840 (n = 1) and one undetermined strain were present. All donor samples had detectable levels of C. difficile from strains F501 (n = 16), P51 (n = 2), P28 (n = 3) and one undetermined strain and all these strains of C. difficile were non-pathogenic. Interestingly, post-FMT samples also had detectable levels of C. difficile from strains F501 (n = 17), P28 (n = 4) and M120 (n = 1). All of these strains were non-pathogenic except one post-FMT sample in which toxigenic strain M120 was detected at a very low relative abundance of 0.05% and this patient had complete resolution of RCDI symptoms. Overall, the relative abundance of majority of C. difficile strains was low (0.6%-3.7%) and it did not make it into the top 25 pre-FMT strains. While our study found higher rates of C. difficile colonization in healthy subjects than other reports, these were non-toxigenic strains which are considered to prevent CDI [29]. It may be emphasized these strains detected in our healthy donor subjects and post-FMT patient samples may not be detected by conventional assays based in clinical practice.

Most of these patients had been on antibiotic therapy (vancomycin, Fidaxomicin) and developed RCDI upon discontinuation of treatment. It has been previously shown that administration of certain antibiotics such as gentamycin, metronidazole, vancomycin, and clindamycin can lead to reduced diversity of the intestinal microbiota and increased susceptibility to intestinal colonization by various pathobionts, such as *C*. *difficile*, which in turn leads to CDI under certain intraluminal environmental conditions [30]. These observations are relevant to our understanding of the pathogenesis of *C*. *difficile* in humans, which under certain clinical conditions can produce toxins A and B, which cause colitis through disruption of tight junctions in the intestinal epithelial barrier after internalization by colonic epithelial cells, and glycosylation of Rho GTPases in colonic epithelial cells [31, 32]. It is noteworthy that 12 of the 22 RCDI patients in our study demonstrated colitis by endoscopic and histopathologic criteria. Administration of FMT to this group of patients with RCDI was associated with a marked resolution of the clinical symptom complex and restoration of the fecal microbiome.

Several GI disorders, including RCDI, IBS, and IBD, as well as antibiotic use, have been associated with bacterial dysbiosis, a compositional and functional alteration in the numerous bacterial communities of the gut microbiome [33]. The DI was significantly higher (p<0·0001) in patients with RCDI before FMT (0·222) than in healthy donors (0·003) and decreased markedly after FMT (p<0·001). In a previous study on the fecal microbiome in cats, Whittemore et al calculated a dysbiosis index after administration of vancomycin and noted similar alterations after antibiotic therapy [34]. We observed a significant inverse correlation between the Shannon index and the DI in patients before and after FMT (Fig 3D). Our study is the first to calculate a DI in RCDI patients at the strain level, although a DI determined at the species or genus level is likely to provide a similar trend. Reduction in the DI of the fecal microbiome in RCDI patients was associated with complete resolution of symptoms following FMT therapy. These salient changes in the microbiome were related to engraftment of bacterial strains from healthy donors to patients with RCDI.

To examine the issue of engraftment of bacterial strains in RCDI patients following FMT, we tracked multiple post-FMT samples in seven patients over varying time periods (Fig 7). We suspected that specific bacterial strains from each donor fecal sample may have the ability to engraft in the intestinal mucosa of the recipient as a result of FMT and restore the microbiota to a healthy state, similar to that of the corresponding donor. Engraftment in our study is a function of the change in a strain’s RA in stool from before to after FMT, along with its RA in the corresponding donor sample. Several factors are likely to play a role in engraftment, including abundance and phylogeny of the bacteria in the donor intestinal microbiota [35]. Among the strains identified in the study, 22 of the 25 top engrafters were from phylum Firmicutes (Fig 7). The top five engrafters, all Firmicutes, were *Anaerostipes hadrus*, *[Ruminococcus] torques L2-14*, *Blautia sp*. *KLE 1732_u_t*, *Dorea formicigenerans ATCC 27755*, *and Lachnocpiraceae bacterium 5_1_63FAA_u_t*. (Fig 8A–8F). These strains need to be cultured and studied in animal models to obtain a deeper understanding of their engraftment and metabolomic properties, though several features of relevance to this study have been described. For example, three of these strong engrafters make the enzymes necessary to ferment non-digestible carbohydrates to produce short-chain fatty acids (SCFA) [36, 37]. Butyrate, the primary SCFA generated by members of phylum Firmicutes, serves as a major energy source for colonocytes in addition to promoting the barrier function of the intestinal epithelium and mediating diverse anti-inflammatory pathways [38–40]. Two key enzymes that enable butyric acid production have been identified: butyrate kinase and BCoAT (Butanoyl-CoA) [38]. Both enzymes are present in *Anaerostipes hadrus*, *Lachnospiraceae bacterium 5_1_63FAA*, and *Dorea formidgenerans ATCC 27755*, three of the top five engrafters found in this study [39]. Identification of these strains is significant, as not all strains of a given species produce SCFAs [40, 41]. In addition to their roles in host nutrition and inflammation, SCFAs downregulate the expression of several virulence genes of known pathobionts [21]. To the best of our knowledge, no other study to date has identified and demonstrated engraftment of these SCFA-producing bacterial strains in RCDI patients following FMT. The mechanism by which FMT from a healthy donor works and restores the gut microbiota is not well understood. Identification of the key bacterial strains and their engraftment may provide better insight into the mechanisms of CDI and development of *C*. *difficile* colitis. Furthermore, these findings may help with the development of the next generation of microbial therapies using specific, well-defined strains that have been purified and grown in vitro to treat a variety of GI disorders associated with bacterial dysbiosis.

### Limitations

Although our study identified a large number of bacterial strains and their potential engraftment, the total number of patients was limited and confined to one institution only. In addition, we were only able to follow a handful of patients for months or years after engraftment. Larger groups of well-identified healthy subjects and RCDI patients from multiple centers, with follow-up data from more patients post-FMT, are needed to confirm and expand these observations. The limitations of shotgun metagenomic sequence analysis is that the CosmosID metagenomic tool classifies genomes to the closest available strain in their reference database. The ability to identify organisms by this algorithm, as with all algorithms, is limited by the contents of its database. It is possible that a strain with a low %unique matches but high %total matches is a closely related taxonomic neighbor not in the database. As many strains exist in nature that have not been sequenced, there’s a distinct possibility that the strain identified is a near neighbor. Further laboratory and animal studies are being planned to confirm the true identity of these bacterial strains by isolation, optimizing anaerobic culture conditions, genomic analysis and by examination of their engraftment properties in humanized mouse models.
